# Single-cell transcriptional profiling revealed the protective effects of Buddleoside in sepsis-associated acute liver injury

**DOI:** 10.3389/fimmu.2025.1713180

**Published:** 2026-01-12

**Authors:** Qingmiao Shi, Na Lou, Leiya Fu, Ying Wang, Chen Xue, Shen Shen, Li Li

**Affiliations:** 1 Department of Infectious Diseases, The First Affiliated Hospital, College of Clinical Medicine, Henan University of Science and Technology, Luoyang, Henan, China; 2 Department of Infectious Diseases, The First Affiliated Hospital of Zhengzhou University, Zhengzhou, Henan, China; 3 Department of Emergency, Henan Provincial People’s Hospital, People’s Hospital of Zhengzhou University, Zhengzhou, Henan, China

**Keywords:** acute liver injury, Buddleoside, endothelial cells, macrophages, neutrophils, sepsis

## Abstract

Sepsis-associated acute liver injury (SALI) results from dysregulated systemic immune responses, ultimately leading to liver dysfunction. Buddleoside (Bud), a naturally derived compound, has exhibited considerable therapeutic potential for liver diseases, which is attributed to its anti-inflammatory, antioxidant, and immunomodulatory effects. This study aims to evaluate the protective effects of Bud in SALI and explore its potential immunomodulatory mechanisms. In this study, SALI was induced in mice using the cecal ligation and puncture model. Biochemical analysis and histopathological evaluation demonstrated that Bud significantly attenuated hepatic inflammation and tissue damage. scRNA-seq analysis revealed that Bud inhibited endothelial cell activation, suppressed the pro-inflammatory phenotype and expression of inflammation-related genes in Ccl4^+^Cxcl1^+^ neutrophils, and decreased cytokine release and inflammation scores in specific macrophage subpopulations. These findings indicate that Bud alleviates SALI by modulating key hepatic cell populations, providing a foundation for the development of natural product-based immunotherapeutic strategies.

## 1 Introduction

Sepsis, a leading cause of mortality in intensive care unit patients, is a systemic inflammatory response syndrome triggered by a dysregulated immune reaction to infection (1). It can rapidly progress to multi-organ dysfunction (2–4). The liver, a vital organ for metabolism and immune regulation, plays a dual role in sepsis (5, 6). On one hand, it supports immune defense by clearing pathogens, secreting acute-phase proteins, and regulating inflammatory mediators. On the other hand, sepsis-associated acute liver injury (SALI) is marked by hypoxic hepatitis resulting from microcirculatory dysfunction, hepatocyte necrosis due to excessive inflammation, and cholestasis caused by bile metabolism disorders (7, 8). Moreover, sepsis-induced damage to the intestinal barrier promotes bacterial and endotoxin translocation, initiating a detrimental intestinal-liver axis that exacerbates liver injury and amplifies systemic inflammation (9, 10). Despite improvements in managing septic symptoms through fluid resuscitation, anti-infection therapy, and organ support, the morbidity and mortality linked to SALI remain high, underscoring its profound impact on patient outcomes (11, 12). The absence of specific therapeutic strategies for SALI highlights the urgent need for novel treatments targeting the pathophysiological mechanisms.

Buddleoside (Bud), a flavonoid compound from the Asteraceae family, demonstrates significant therapeutic potential for liver diseases due to its anti-inflammatory, antioxidant, and immunomodulatory effects (13, 14). Studies show that Bud activates the autophagy-lysosomal pathway through the AMPK-TFEB axis in the liver, offering protective effects in mouse models of nonalcoholic steatohepatitis (15). In carbon tetrachloride (CCl4)-induced acute liver injury models, Bud alleviates hepatic damage by modulating the TLR4/MAPK/Nrf2 signaling pathway, which reduces inflammation, alleviates oxidative stress, and enhances autophagy (16). Additionally, Bud in combination with Coptis chinensis inflorescence extract has been shown to reduce CCl4-induced HepG2 cell injury *via* the MAPK/Keap1-Nrf2 signaling pathway (17). Bud also inhibits TNF-α-induced hepatocyte apoptosis, mitigating liver injury and delaying liver failure progression (18). However, the precise role and underlying molecular mechanisms of Bud in SALI remain unclear, particularly regarding its protective effects through immunoregulatory pathways, which warrant further exploration.

In recent years, single-cell RNA sequencing (scRNA-seq) has emerged as a powerful tool for examining gene expression profiles at the single-cell level, enabling precise characterization of cellular heterogeneity within liver tissue (19). This technology offers a novel approach to unraveling the molecular mechanisms of SALI and exploring the potential role of Bud in its treatment. scRNA-seq studies have identified several macrophage populations in the liver that play critical immunomodulatory roles in SALI, contributing to both liver injury and repair (20, 21). One study observed a reduction in hepatic endothelial cells (ECs) during SALI, while macrophages were recruited and contributed to liver inflammation through chemokine and inflammatory cytokine secretion. Simultaneously, abnormal neutrophil infiltration and lymphocyte apoptosis led to immunosuppression, suggesting these cell populations as potential therapeutic targets (22, 23). Additionally, scRNA-seq analyses have pinpointed specific hepatic endothelial and neutrophil subsets associated with acute liver dysfunction during sepsis progression (24). Collectively, these findings highlight the value of integrating single-cell transcriptomic analysis with functional studies to identify key cellular targets and regulatory signaling pathways modulated by Bud in SALI. This approach could uncover its immunoregulatory mechanisms and inform future therapeutic investigations.

Building on this foundation, the present study aims to systematically assess the protective effects of Bud in SALI and explore its underlying immune-regulatory mechanisms through single-cell transcriptomic approaches. The findings are anticipated to address existing research gaps regarding Bud in the context of SALI and provide a theoretical basis for the development of natural product-based immunotherapies.

## 2 Materials and methods

### 2.1 Experimental animals

Male C57BL/6 mice, aged 6–8 weeks, were purchased from Beijing Vital River Laboratory Animal Technology Co., Ltd. (Beijing, China). Prior to the experiment, the mice were acclimatized for one week in a specific-pathogen-free facility maintained at 22 ± 2°C, under a 12-hour light/dark cycle, with unrestricted access to food and water. All experimental procedures were conducted in compliance with laboratory animal care guidelines and were approved by the Ethical Committee of the First Affiliated Hospital of Zhengzhou University (Ethics approval number. 2025-KY-0431-001).

### 2.2 SALI animal model construction

The cecal ligation and puncture (CLP) procedure in rodents has become the most widely used model for experimental sepsis. It is recognized for its ability to accurately replicate the pathophysiological processes of polymicrobial sepsis in humans and is currently considered the gold standard in sepsis research. Therefore, sepsis was induced in the mice using the CLP model in this study (25). Briefly, the mice were anesthetized by intraperitoneal administration of pentobarbital sodium (50 mg/kg). The cecum was ligated with sutures 1 cm from the blind end, punctured once with a 21-gauge needle, and a small amount of intestinal content was extruded. The cecum was then repositioned, and the incision was closed in layers.

### 2.3 Reagents

Bud (CAS number: 480-36-4, Catalog number: B20860) and silymarin (CAS number: 22888-70-6, Catalog number: B21185) was provided by Shanghai Yuanye Biotechnology Co., Ltd. Bud and silymarin were individually dissolved in dimethyl sulfoxide (DMSO) to prepare stock solutions, which were then diluted with normal saline to the desired working concentration for administration.

### 2.4 Animal grouping and treatment

Experiment 1: Following one week of acclimatization, thirty-six C57BL/6 mice were randomly assigned to the following six groups. Control group (n=6): laparotomy and cecum exposure without ligation or puncture. Model group (n=6): mice received daily intraperitoneal injections of 200 μL of vehicle solution for three days and were subsequently subjected to CLP operation. Bud-L, Bud-M, and Bud-H group (n=6 per group): mice were administered daily intraperitoneal injections of low-dose (15 mg/kg), medium-dose (30 mg/kg), or high-dose (60 mg/kg) Bud solution, respectively, for three consecutive days prior to CLP surgery. Silymarin group (n=6): mice received daily intraperitoneal injections of the positive control drug silymarin at a dose of 100 mg/kg for three days and were subsequently subjected to CLP operation. Twenty-four hours post-CLP, the mice were euthanized via intraperitoneal injection of an excessive dose of pentobarbital sodium (150 mg/kg). Subsequently, blood samples and fresh liver tissues were collected from each mouse. Serum biochemical markers were analyzed, and liver tissues were fixed in 4% paraformaldehyde for histopathological evaluation.

Experiment 2: Following one week of acclimatization, twelve C57BL/6 mice were randomly assigned to the following groups. CLP group (n=6), the mice received daily intraperitoneal injections of 200 μL of vehicle solution for three days and were subjected to CLP operation. Bud group (n=6), the mice received Bud solution at a high-dose of 60 mg/kg for three days and were subjected to CLP operation. Twenty-four hours post-CLP, the mice were euthanized via intraperitoneal injection of an excessive dose of pentobarbital sodium (150 mg/kg). Then, fresh liver tissues were collected and used to prepare single-cell suspensions for subsequent scRNA-seq.

### 2.5 Serum biochemical examination

Serum was isolated by centrifuging blood samples at 4000 g for 20 minutes, according to the manufacturer’s instructions. Alanine aminotransferase (ALT) and aspartate transaminase (AST) levels in the supernatant were quantified using a dry chemistry analyzer.

### 2.6 Histopathological analysis

Liver tissue samples were fixed in 4% paraformaldehyde for 24 hours, followed by dehydration with graded ethanol solutions and clearing in xylene. The tissues were then embedded in paraffin and sectioned into 5 μm-thick slices. Paraffin sections were deparaffinized, rehydrated, and stained with hematoxylin-eosin (H&E). The stained sections were digitized using the Pannoramic 3DHISTECH imaging system and analyzed with CaseViewer software.

### 2.7 Immunofluorescence staining of tissue sections

IF was conducted on 4-μm-thick liver sections. After deparaffinization, rehydration, and antigen retrieval, the sections were blocked with 5% bovine serum albumin (BSA). Primary antibodies, including CD31 (1:300, #80530-1-RR, Proteintech), CD11b (1:2000, #ab133357, Abcam), Ly6G (1:300, #87048, Cell Signaling Technology), and F4/80 (1:2000, #28463-1-AP, Proteintech), were applied. For multiplex IF (mIF), a tyramide signal amplification (TSA) protocol was used. Following incubation with primary antibodies, the sections were treated with HRP-conjugated secondary antibody, followed by fluorophore-conjugated tyramide. Excess antibodies were washed off, and nuclei were counterstained with DAPI. Fluorescence signals were visualized using the Pannoramic 3DHISTECH scanner.

### 2.8 Acquisition of scRNA-seq dataset

Our previous study analyzed the single-cell RNA profiling of liver tissue following sepsis-induced acute liver injury and TPPU treatment (26). The scRNA-seq dataset GSE290684, which includes scRNA-seq data from six liver samples collected from vehicle-treated SALI mice in the prior study, was incorporated into the present analysis and designated as the CLP group.

### 2.9 scRNA-seq of in-house data

Six fresh liver tissues from high-dose Bud-treated mice were rinsed with cold PBS and minced into approximately 0.5 mm^3^ pieces. To minimize cell death, all tissues were kept on ice throughout the procedure. Tissue digestion was performed using the Mouse Liver Dissociation Kit (Miltenyi Biotec, Germany) according to the manufacturer’s instructions, followed by incubation at 37°C for 30 minutes with gentle agitation. The resulting cell suspension was filtered through a 70 μm cell strainer into a sterile tube and centrifuged at 300 × g for 5 minutes. The supernatant was discarded, and the cell pellet was treated with erythrocyte lysis buffer. After a second centrifugation step, the pellet was resuspended in PBS supplemented with 0.04% BSA to generate a single-cell suspension suitable for scRNA-seq.

Single-cell suspensions were loaded onto the MobiNova-100 platform (MobiDrop, Zhejiang, China). Cells were mixed with barcoded gel beads and partitioning oil to generate single-cell water-in-oil droplets. Within these droplets, mRNA was captured by oligo-dT beads, reverse transcribed into cDNA, and the cDNA was amplified *via* PCR. Libraries were constructed through fragmentation, end repair, adapter ligation, and size selection. The qualified libraries were then sequenced on the MobiNova-100 system, targeting ≥50,000 reads per cell for robust gene detection.

### 2.10 scRNA-seq data processing

Following quality control of the raw sequencing data, the gene expression matrix was constructed by aligning the sequences to the mouse reference genome using Cell Ranger. Normalization and clustering analysis were conducted using the Seurat R package, and the UMAP algorithm was employed for dimensionality reduction and visualization. Using predefined gene sets (**Supplementary Tables 1, 2**), the gene set variation analysis (GSVA) algorithm was applied to calculate inflammatory response and cytokine scores (27, 28). Differentially expressed genes (DEGs) were identified based on thresholds of |log2 (fold change)| > 0.25 and p-value < 0.05. Pseudotime trajectory analysis was conducted using the Monocle 2 software package, and the CellChat tool was utilized to analyze the intercellular communication network (29, 30).

### 2.11 Statistical analysis

Statistical analysis was performed using R software or GraphPad Prism. Comparisons of continuous variables between two groups were conducted using the unpaired Student’s t-test, while comparisons across multiple groups were carried out using One-way ANOVA or Welch’s ANOVA, as appropriate. A p-value of less than 0.05 was considered statistically significant.

## 3 Results

### 3.1 Bud alleviated CLP-induced SALI

**Figure 1:**
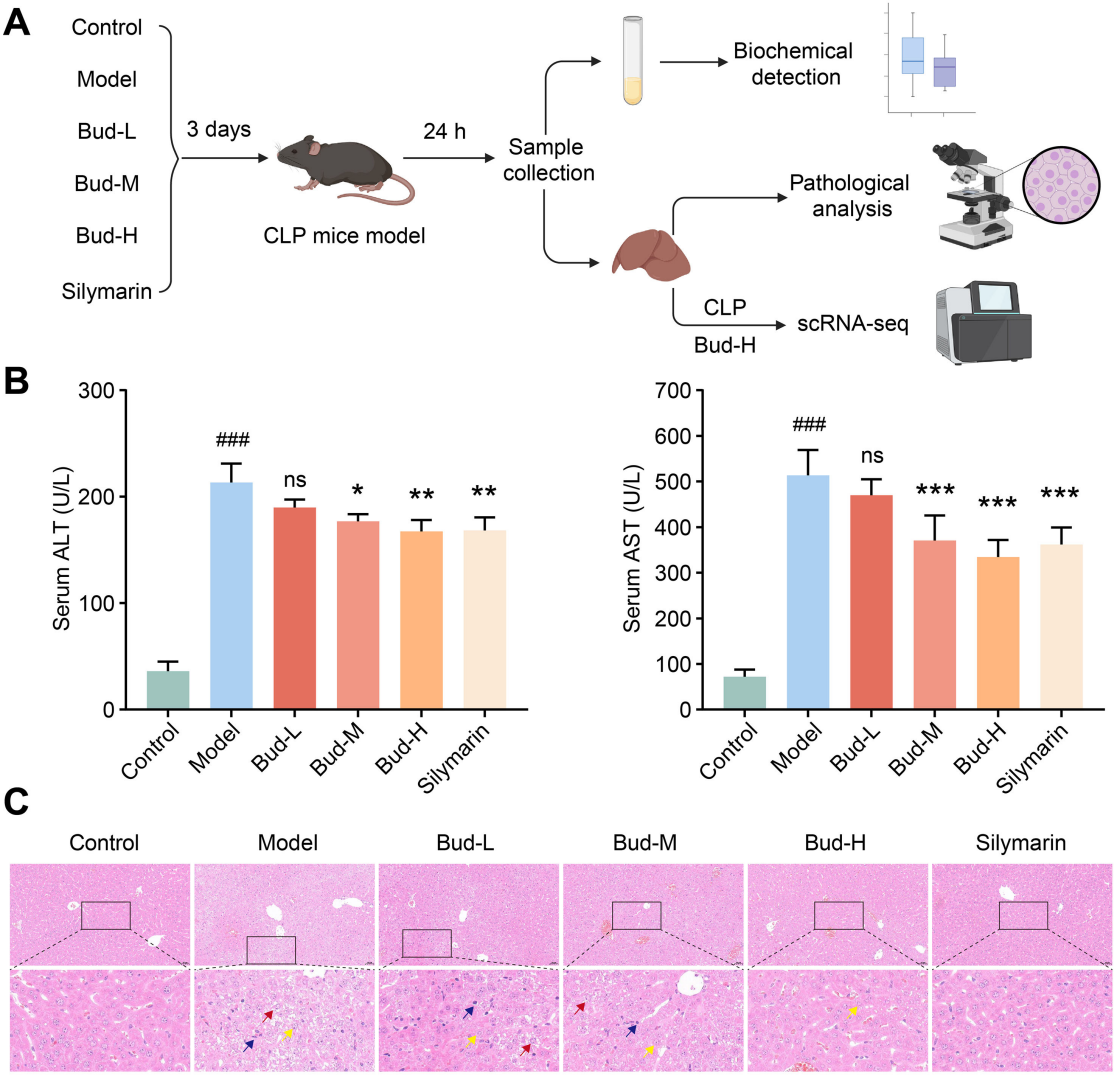
Bud alleviates CLP-induced SALI. **(A)** Experimental workflow. **(B)** Serum ALT and AST levels in each group. **(C)** (Upper) Representative H&E images of each group. Scale bars: 50 μm. (Below) The enlarged H&E image. The yellow arrow indicates hydropic degeneration, the red arrow indicates ballooning degeneration, and the blue arrow indicates inflammatory cell. Compared with the Control group, ^###^p < 0.001; compared with the Model group, *p < 0.05, **p < 0.01, ***p < 0.001; ns, nonsignificant.

To investigate the protective effects of Bud in SALI, a CLP-induced murine model of SALI was established. Thirty-six mice were randomly assigned to Control, Model, Bud-L, Bud-M, Bud-H, or Silymarin group, with treatment administered three days prior to CLP surgery. Blood and liver samples were collected 24 hours post-surgery for subsequent analysis (**Figure 1A**). Liver injury was assessed using biomarkers such as serum ALT and AST levels, as well as H&E staining. Compared with the Control group, the Model group exhibited significantly elevated serum levels of ALT and AST (**Figure 1B**). Following medium-dose Bud, high-dose Bud, and silymarin treatment, the ALT and AST levels were effectively reversed (**Figure 1B**). Furthermore, H&E staining revealed more disorganized hepatic lobular structures, increased hepatocyte swelling and degeneration, and greater inflammatory cell infiltration in the Model group. Bud and silymarin treatment partially improved hepatic lobular organization, preserved hepatocyte morphology, and reduced inflammatory infiltration (**Figure 1C**). These results collectively suggest that Bud alleviated CLP-induced SALI by reducing hepatocyte damage and suppressing the inflammatory response.

### 3.2 scRNA-seq revealed heterogeneous cell subpopulations and their responses to Bud administration

**Figure 2:**
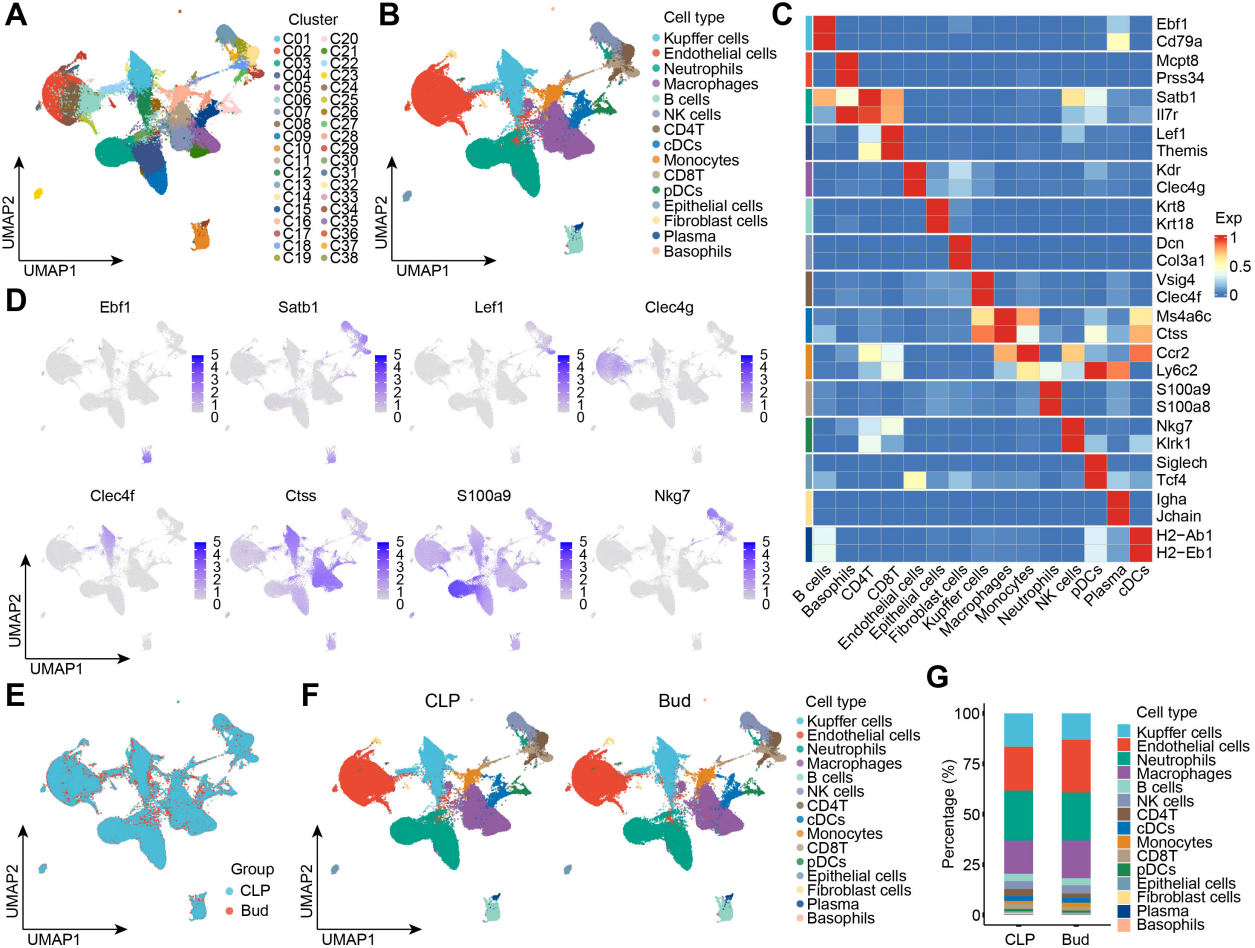
scRNA-seq reveals heterogeneous cell subpopulations and their responses to Bud administration. **(A)** UMAP visualizing 27 cell subpopulations. **(B)** UMAP illustrating 15 functional cell types. **(C)** Heatmap showing differentially expressed genes across cell types. **(D)** Single-cell expression patterns of marker genes for specific cell types. **(E)** UMAP depicting unsupervised cell clustering of total samples. **(F)** UMAP showing differential cell composition between CLP and Bud groups (n = 6 per group). **(G)** Stacked bar exhibiting the percentage of each cell type in CLP and Bud groups.

To evaluate the effect of high-dose Bud on the hepatic cellular landscape in SALI, twelve C57BL/6 mice were randomly assigned to the CLP or Bud group. Following three days of continuous treatment, the SALI model was induced *via* CLP, and fresh liver tissues were collected 24 hours post-surgery for scRNA-seq analysis. UMAP-based dimensionality reduction visualized 38 distinct subpopulations of cells, including basophils, B cells, plasma cells, T cells, neutrophils, Kupffer cells (KCs), macrophages, natural killer (NK) cells, dendritic cells (DCs), monocytes, ECs, epithelial cells, and fibroblasts (**Figures 2A, B**). The identity of each cluster was confirmed by analyzing the expression of corresponding marker genes (**Figure 2C**). Classic marker genes such as E*b*f1 in B cells, *Satb1* in CD4^+^ T cells, *Lef1* in CD8^+^ T cells, *Clec4f* in KCs, *Ctss* in macrophages, *S100a9* in neutrophils, *Nkg7* in NK cells, and *Clec4g* in ECs were prominently expressed (**Figures 2C, D**). Comparative analysis between the CLP and Bud groups revealed alterations in the cellular composition of the hepatic microenvironment (**Figures 2E–G**). Bud administration notably altered the proportions of several key cell types, with ECs, macrophages, and neutrophils being the most prominent populations. Overall, scRNA-seq effectively captured transcriptional heterogeneity and confirmed significant differences in cellular composition between the CLP and Bud groups, providing crucial transcriptomic evidence for analyzing changes in macrophages, neutrophils, and ECs in SALI at the single-cell level.

### 3.3 Bud regulated the heterogeneity and functional transcription profiles of ECs

**Figure 3:**
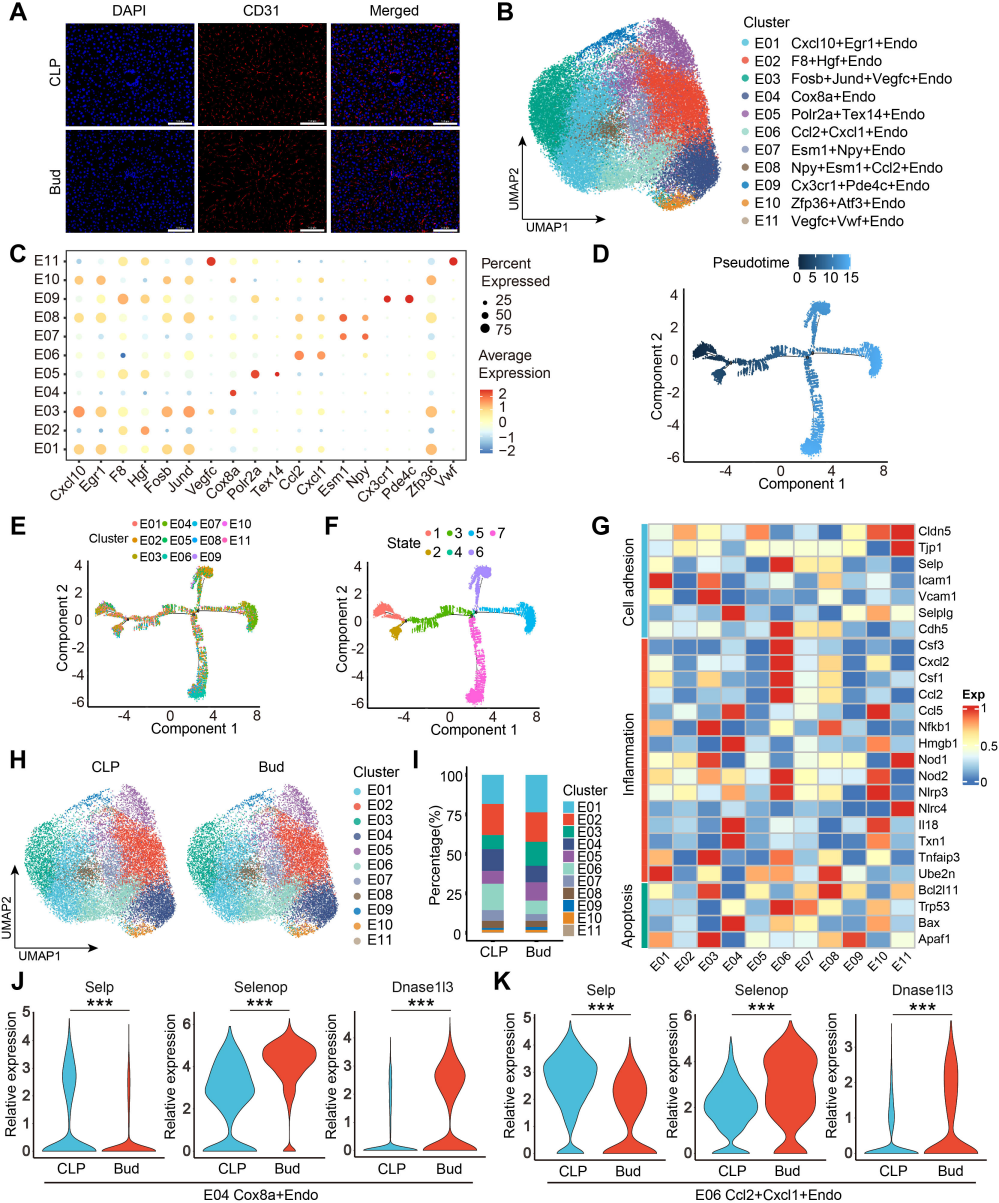
Bud regulates the heterogeneity and functional transcription profiles of ECs. **(A)** Representative mIHC images showing the localization of CD31-positive cells (ECs). Scale bar: 100 μm. **(B)** Unsupervised clustering identifies 11 distinct EC subpopulations. **(C)** Cluster-specific marker genes defining EC types. **(D-F)** Pseudotime trajectory of EC development, with colors representing pseudotime, subtypes, and states. **(G)** Expression profiles of cell adhesion, inflammation, and apoptosis markers distinguishing functional states. **(H)** Distribution of ECs in CLP and Bud groups. **(I)** Subpopulation percentages in CLP and Bud groups. **(J, K)** Gene expression levels of *Selp*, *Selenop*, and *Dnase1l3* in Cox8a^+^Endo and Ccl2^+^Cxcl1^+^Endo subpopulations. ***p < 0.001.

Hepatic sinusoidal ECs form a critical filtering barrier between hepatocytes and the bloodstream, playing a central role in maintaining hepatic homeostasis by regulating vascular tone, inflammatory responses, and immune function (31). mIF confirmed the *in-situ* localization of CD31-positive ECs (**Figure 3A**). Further scRNA-seq analysis was conducted to explore the impact of Bud on EC heterogeneity and functional alterations. The UMAP algorithm identified 11 distinct EC subpopulations, each characterized by a unique marker gene signature (**Figures 3B, C**). Pseudotime trajectory analysis revealed a hierarchical differentiation pattern, with each subpopulation labeled in different colors, originating from a common root and diverging into three major branches (**Figures 3D–F**). Notably, the Cxcl10^+^Egr1^+^Endo subpopulation was primarily located at the initial stage, suggesting it may represent the origin of EC differentiation. Functional characteristics of ECs were examined using a heatmap, which displayed gene expression patterns linked to cell adhesion, inflammation, and apoptosis (**Figure 3G**). The Cxcl10^+^Egr1^+^Endo subpopulation showed high expression of the cell adhesion-related gene *Icam1*. The Cox8a^+^Endo, Ccl2^+^Cxcl1^+^Endo, and Zfp36^+^Atf3^+^Endo subpopulations exhibited pronounced inflammatory features, with significant enrichment of apoptosis-related genes such as *Bax*, *Trp53*, and *Apaf1*, respectively.

Following Bud administration, significant changes in EC subpopulation composition were observed, particularly a marked reduction in the percentage of Cox8a^+^Endo and Ccl2^+^Cxcl1^+^Endo (**Figures 3H, I**). Differential gene expression analysis revealed that *Selp* was significantly downregulated in these subpopulations after Bud treatment, while *Selenop* and *Dnase1l3* were notably upregulated (**Figures 3J, K**). *Selp*, expressed in activated ECs, plays a key role in mediating leukocyte rolling and adhesion, which are essential steps in the inflammatory response (32, 33). The N-terminal selenomethionine of *Selenop* exerts antioxidant effects, maintaining intracellular redox homeostasis and protecting against oxidative stress-induced damage (34). Dnase1l3 is involved in degrading neutrophil extracellular traps (NETs), thereby reducing NET-mediated organ damage (35). The downregulation of *Selp* suggests that Bud effectively suppresses EC activation, limiting leukocyte recruitment and infiltration into damaged liver tissue, thereby alleviating inflammation and secondary tissue injury. Upregulation of *Selenop* enhances the clearance of reactive oxygen species, preserving the structural and functional integrity of ECs. Increased expression of *Dnase1l3* indicates that Bud enhances ECs’ ability to eliminate NETs, reducing NET-mediated cytotoxicity and chronic inflammation. Collectively, these results demonstrate that Bud modulates EC composition and function, playing a pivotal role in maintaining hepatic microenvironmental homeostasis, mitigating inflammatory responses, and protecting against hepatic tissue damage.

### 3.4 Bud inhibited the inflammatory state of Ccl4^+^Cxcl1^+^Neu to mitigate SALI

**Figure 4:**
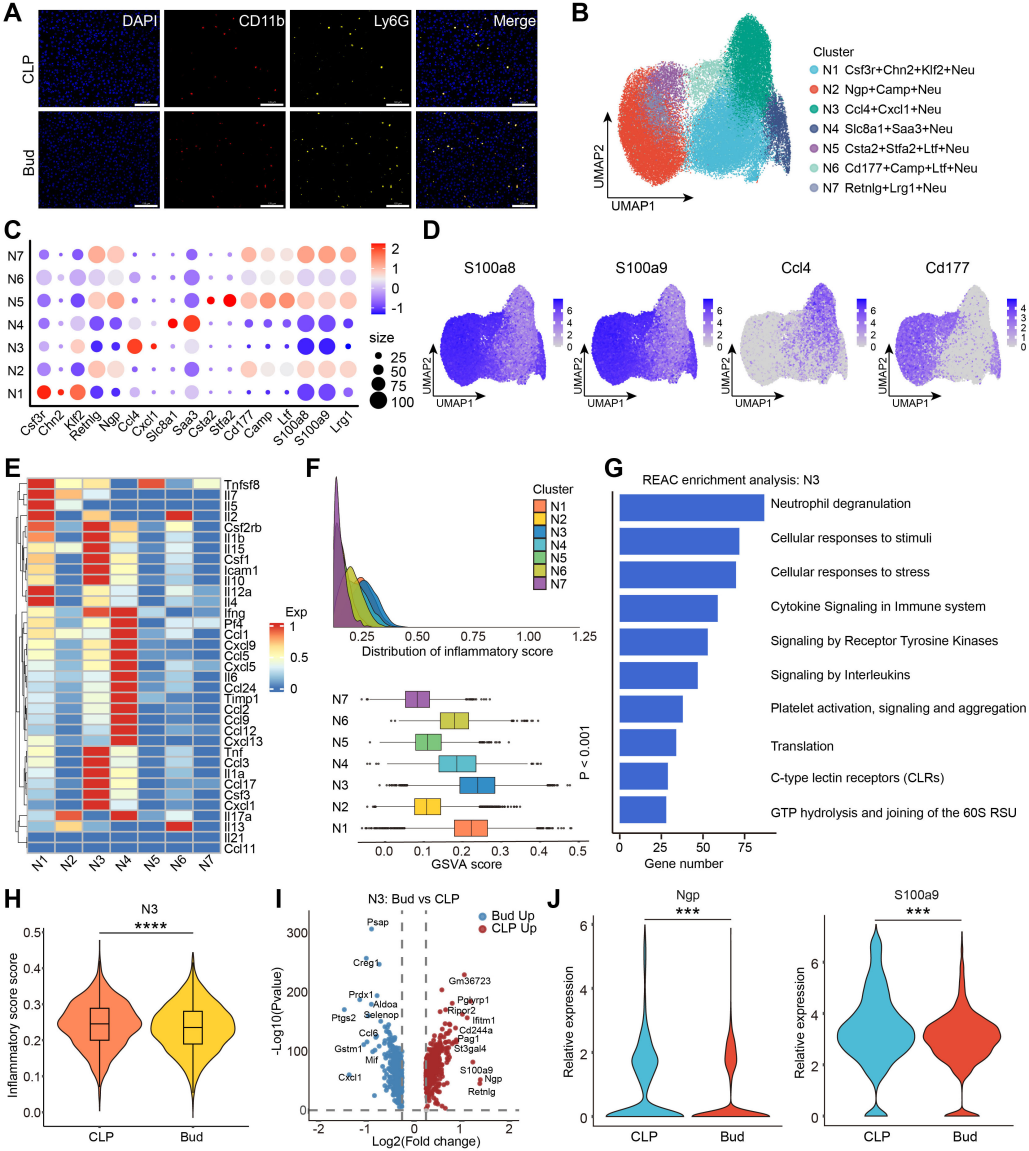
Bud inhibits the inflammatory state of Ccl4^+^Cxcl1^+^Neu to mitigate SALI. **(A)** Representative mIHC images showing the localization of CD11b^+^Ly6G^+^ cells (neutrophils) in CLP group and Bud group. Scale bar: 100 μm. **(B)** UMAP of 7 neutrophil subpopulations. **(C)** Dot plot of cluster-specific gene expression for neutrophil subpopulations. **(D)** UMAP of marker genes in neutrophils. **(E)** Heatmap showing pro- and anti-inflammatory gene expression across 7 neutrophil subpopulations. **(F)** Inflammation scores of 7 neutrophil subpopulations. **(G)** Reactome enrichment analysis of N03 (Ccl4^+^Cxcl1^+^Neu) subpopulation. **(H)** Violin plot of inflammation scores in N03 subpopulation (Ccl4^+^Cxcl1^+^Neu) between CLP and Bud groups. **(I)** Volcano plot of differentially expressed genes in N03 subpopulation (Ccl4^+^Cxcl1^+^Neu). **(J)** Violin plot of *Ngp* and *S100a9* expression levels in N03 subpopulation (Ccl4^+^Cxcl1^+^Neu). ***p < 0.001, ****p < 0.0001.

Emerging studies suggest that hyperactivation of neutrophils contributes to tissue damage by inducing ECs to adopt a pro-inflammatory and procoagulant phenotype, thus increasing vascular permeability (36). mIF confirmed the *in-situ* localization of CD11b^+^Ly6G^+^ neutrophils (**Figure 4A**). Using canonical marker genes expressed in neutrophils, unsupervised clustering of the neutrophil transcriptome identified seven distinct subpopulations, as revealed by UMAP analysis, highlighting neutrophil heterogeneity in the liver microenvironment of SALI (**Figures 4B–D**). To examine the functional differences among these subpopulations, a heatmap was generated to visualize the differential expression patterns of anti-inflammatory and pro-inflammatory gene sets (**Figure 4E**). Notably, pro-inflammatory genes were expressed at low levels in the N02 (Ngp^+^Camp^+^Neu), N05 (Csta2^+^Stfa2^+^Ltf^+^Neu), N06 (Cd177^+^Camp^+^Ltf^+^Neu), and N07 (Retnlg^+^Lrg1^+^Neu) subpopulations, whereas they were highly expressed in the N01 (Csf3r^+^Chn2^+^Klf2^+^Neu), N03 (Ccl4^+^Cxcl1^+^Neu), and N04 (Slc8a1^+^Saa3^+^Neu) subpopulations. Inflammation scores were calculated to quantitatively assess the inflammatory state of each neutrophil subpopulation (**Figure 4F**). The Ccl4^+^Cxcl1^+^Neu subpopulation exhibited the highest inflammation scores among all identified neutrophil subpopulations. Reactome enrichment analysis showed that genes highly expressed in the Ccl4^+^Cxcl1^+^Neu subpopulation were significantly enriched in inflammation-related pathways, including neutrophil degranulation, cellular responses to stress, cytokine signaling, and receptor tyrosine kinase signaling (**Figure 4G**).

A violin plot comparing inflammation scores in the Ccl4^+^Cxcl1^+^Neu subpopulation between the CLP and Bud groups demonstrated that Bud administration significantly suppressed the inflammatory activity of this subpopulation (**Figure 4H**). Further analysis of DEGs in the Ccl4^+^Cxcl1^+^Neu subpopulation using a volcano plot revealed substantial transcriptional alterations (**Figure 4I**). Previous studies have shown that S100a9, secreted extracellularly under inflammatory conditions, promotes neutrophil degranulation, NET formation, and neutrophil-EC adhesion (37, 38). Notably, Bud treatment significantly downregulated S100a9 expression in the Ccl4^+^Cxcl1^+^Neu subpopulation, suggesting that Bud may mitigate inflammatory responses (**Figure 4J**). Additionally, downregulation of neutrophil granule protein (Ngp) was associated with a reduced release of cytotoxic enzymes such as cathepsin G and elastase, thereby inhibiting degranulation activity (**Figure 4J**). Collectively, these results demonstrate that Bud mitigates SALI by inhibiting the inflammatory function of the Ccl4^+^Cxcl1^+^Neu subpopulation.

### 3.5 Bud modulated macrophage subpopulation heterogeneity to inhibit liver injury

**Figure 5:**
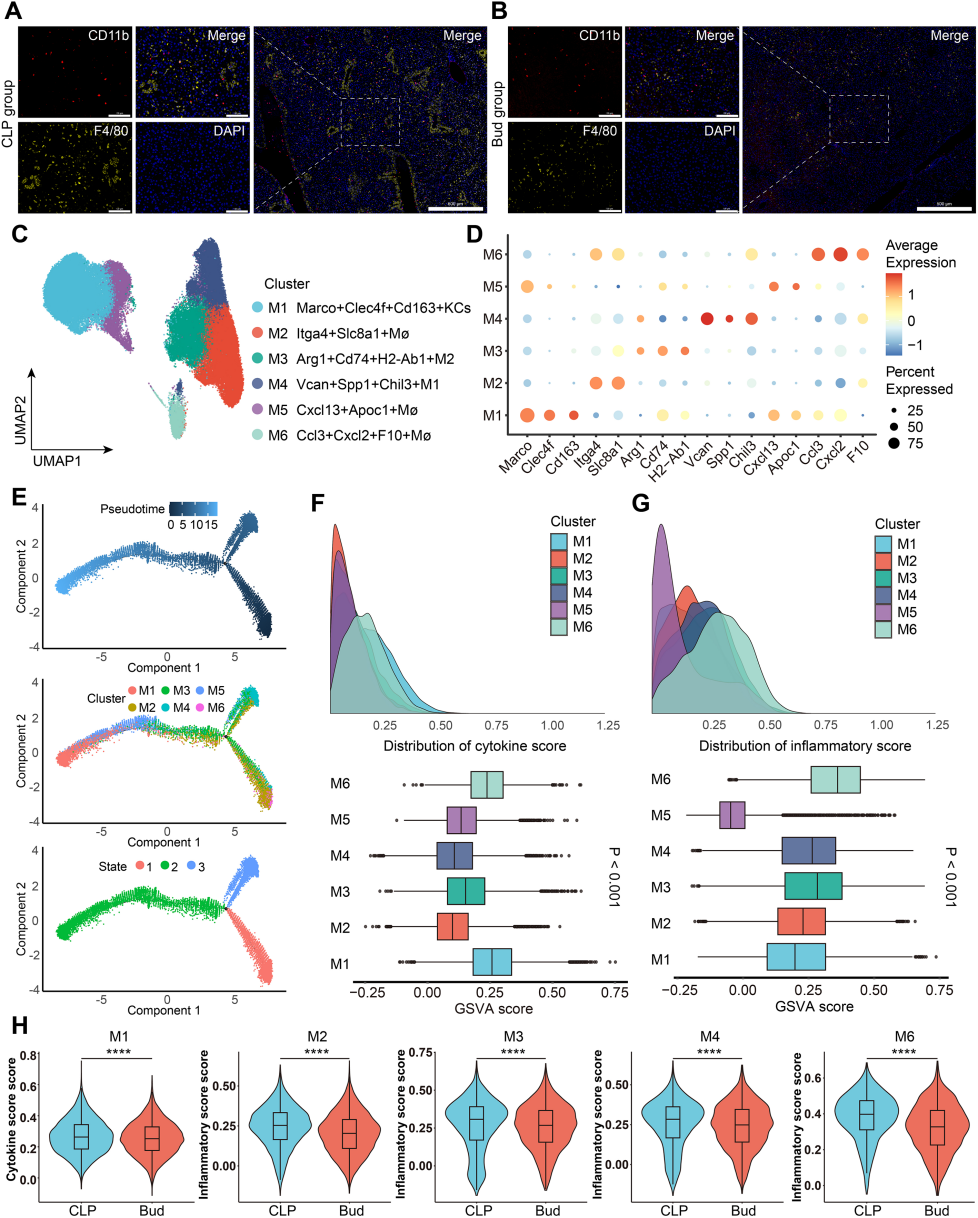
Bud modulates macrophage subpopulation heterogeneity to inhibit liver injury. **(A, B)** Representative mIHC images showing the localization of CD11b^+^F4/80^+^ cells (macrophages) in the liver tissues of **(A)** CLP group, **(B)** Bud group. Scale bar: 500 μm or 100 μm. **(C)** UMAP of 7 macrophage subpopulations. **(D)** Dot plot of cluster-specific gene expression for macrophages. **(E)** Pseudotime trajectory of macrophage development, with colors representing pseudotime, subtypes, and states. **(F)** Cytokine scores of 6 macrophage subpopulations. **(G)** Inflammation scores of 6 macrophage subpopulations. **(H)** Violin plot showing cytokine and inflammation scores in specific macrophage subpopulations. ****p < 0.0001.

Accumulating evidence indicates that macrophages play a significant role in the injury and repair of the liver. mIF confirmed the *in-situ* localization of CD11b^+^F4/80^+^ macrophages in the liver tissues of CLP group and Bud group (**Figures 5A, B**). To characterize the heterogeneity and functional diversity of macrophages, unsupervised clustering analysis was performed on scRNA-seq data derived from macrophages. Six distinct macrophage subpopulations were identified based on specific marker gene expression profiles (**Figures 5C, D**). The M01 (Marco^+^Clec4f^+^Cd163^+^KCs) subpopulation showed high expression of classical liver-resident macrophage markers, *Macro* and *Clec4f*. The M2-like macrophage markers, *Arg1* and *H2-Ab1*, were predominantly enriched in the M03 (Arg1^+^Cd74^+^H2-Ab1^+^Mø) subpopulation, while the M1-like markers, *Vcan* and *Chil3*, were upregulated in the M04 (Vcan^+^Spp1^+^Chil3^+^Mø) subpopulation. Additionally, the M05 (Cxcl13^+^Apoc1^+^Mø) and M06 (Ccl3^+^Cxcl2^+^F10^+^Mø) subpopulations were characterized by the expression of cytokine-associated genes, such as *Cxcl13* and *Ccl3*. The Monocle 2 algorithm was then used to explore the potential differentiation trajectories of macrophages. The six subpopulations were assigned to three distinct differentiation states, with Ccl3^+^Cxcl2^+^F10^+^Mø likely representing the initial differentiation state, while Marco^+^Clec4f^+^Cd163^+^KCs and Cxcl13^+^Apoc1^+^Mø were identified as the terminal differentiation states (**Figure 5E**). Cytokine and inflammation scores were quantified for each subpopulation using GSVA analysis (**Figures 5F, G**). The Marco^+^Clec4f^+^Cd163^+^KCs and Ccl3^+^Cxcl2^+^F10^+^Mø subpopulations exhibited higher cytokine scores, while the Cxcl13^+^Apoc1^+^Mø subpopulation had the lowest inflammation score. Comparative analysis between the CLP and Bud groups revealed a significant reduction in the cytokine score of the Marco^+^Clec4f^+^Cd163^+^KCs subpopulation in the Bud group (**Figure 5H**). Furthermore, the inflammation scores of the Itga4^+^Slc8a1^+^Mø, Arg1^+^Cd74^+^H2-Ab1^+^Mø, Vcan^+^Spp1^+^Chil3^+^Mø, and Ccl3^+^Cxcl2^+^F10^+^Mø subpopulations were notably decreased following Bud administration (**Figure 5H**). Collectively, these results suggest that Bud exerts protective effects by inhibiting cytokine secretion and inflammatory activation in specific macrophage subpopulations.

### 3.6 Bud suppressed CXCL2-CXCR2 signaling transduction between ECs and neutrophils

**Figure 6:**
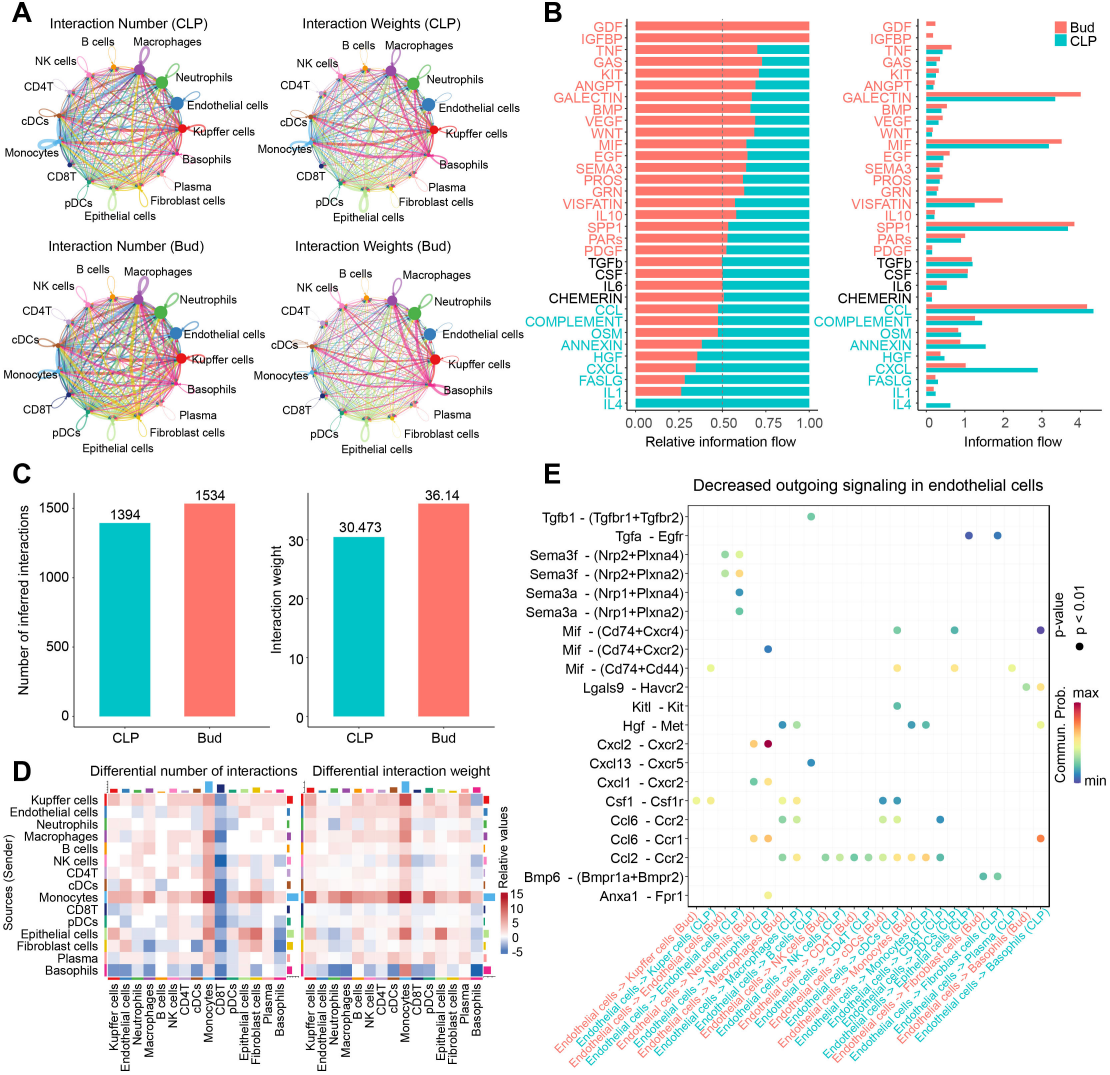
Bud suppresses CXCL2-CXCR2 signaling between ECs and neutrophils. **(A)** Circle plot visualizing the number and weight of cell-cell communications across different cell types. **(B)** Bar plot showing relative information flow between CLP and Bud groups. **(C)** Bar plot quantifying the number and weight of cell-cell communication between CLP and Bud groups. **(D)** Heatmap depicting the differential number and weight of cell-cell communication between CLP and Bud groups. **(E)** Bubble plot exhibiting decreased outgoing ligand-receptor signaling in ECs.

To analyze intercellular communication across different cell types in the CLP and Bud groups, cell-cell communication networks were constructed based on single-cell transcriptional profiles and visualized using circle plots (**Figure 6A**). Each cell type engaged in extensive communication through multiple ligand-receptor interactions, indicating the presence of complex intercellular signaling networks in both groups. Further analysis of the information flow and relative information flow revealed that Bud modulates intercellular communication within the hepatic microenvironment (**Figure 6B**), potentially influencing key signaling pathways involved in inflammation and immune cell recruitment. Notably, the information flow in chemokine-related pathways, including the CCL and CXCL families, was significantly reduced in the Bud group, suggesting that Bud may inhibit the chemotactic activity of immune cells.

Additionally, the total number of intercellular interactions increased from 1,394 in the CLP group to 1,534 in the Bud group, with the interaction weight rising from 30.473 to 36.14 (**Figure 6C**). Heatmap visualization further demonstrated a general decrease in interactions between ECs and other cells in the Bud group compared to the CLP group (**Figure 6D**). Subsequently, ligand-receptor pair alterations in ECs were examined following Bud administration. The receptors for CXCL2 include ACKR1 (atypical chemokine receptor 1) and CXCR2 (C-X-C chemokine receptor 2), with CXCR2 being the primary functional receptor (39). The CXCL2-CXCR2 signaling axis was significantly downregulated between ECs and neutrophils after Bud treatment (**Figure 6E**). Given prior evidence that CXCR2 antagonists (e.g., SB225002 and AZD5069) reduce neutrophil infiltration in the liver, the downregulation of the CXCL2-CXCR2 axis may underlie Bud’s inhibitory effect on neutrophil recruitment (40, 41).

## 4 Discussion

Bud has garnered widespread recognition for its diverse pharmacological activities, including anti-inflammatory, cardiovascular protective, and metabolic regulatory properties, and has attracted growing interest for its potential role in hepatic inflammatory diseases (14, 42). This study aimed to investigate the protective effects of Bud against SALI and elucidate its underlying immunoregulatory mechanisms through single-cell transcriptomic profiling. The findings revealed that Bud significantly alleviated hepatic inflammation and tissue damage. The underlying mechanisms likely involve alterations in the composition and functional reprogramming of key cell populations, specifically ECs, neutrophils, and macrophages. Notably, Bud suppressed EC activation, inhibited the pro-inflammatory phenotype and expression of inflammation-related genes in the Ccl4^+^Cxcl1^+^Neu subpopulation, and reduced cytokine secretion and inflammation scores in specific macrophage subpopulations. These results provide compelling evidence supporting Bud as a promising therapeutic agent for SALI.

It is essential to contextualize the findings of this study within our previous work, which examined the immunoregulatory mechanism of the soluble epoxide hydrolase (sEH) inhibitor TPPU in ameliorating SALI (26). Our prior research on TPPU primarily demonstrated its role in promoting the expansion of anti-inflammatory CD206^+^CD73^+^ M2-like macrophages and PDL1^-^CD39^-^CCR2^+^ neutrophils, as well as reprogramming liver neutrophils toward an anti-inflammatory CAMP^+^NGP^+^CD177^+^ phenotype. The present study reveals that the natural flavonoid Bud exerts its protective effects through a fundamentally distinct mechanism, identifying Ccl4^+^Cxcl1^+^ neutrophils as a previously underappreciated cellular target in SALI and demonstrating that Bud potently suppresses their pro-inflammatory activation. Collectively, TPPU and Bud represent two non-overlapping classes of immunomodulators that converge in protecting the liver during sepsis through divergent mechanisms. This distinction not only highlights the multifaceted nature of immune dysregulation in SALI but also positions Bud as a unique, naturally derived therapeutic candidate with a mode of action independent of sEH inhibition.

Bacterial infection-induced sepsis triggers a systemic inflammatory response, with the liver being a primary target of damage (5). Bacterial components and their toxins contribute to hepatic inflammation and dysfunction through mechanisms such as gut dysbiosis, bacterial translocation, and activation of inflammatory signaling pathways (43, 44). Among these, bacterial translocation from the gut has emerged as a critical initiating event. Gong et al. reported that sepsis disrupts the intestinal barrier, promoting bacterial translocation, while fecal microbiota transplantation from sepsis-resistant mice mitigates liver injury (45). This finding directly supports the regulatory role of the gut microbiota in determining susceptibility to hepatic injury. Following disruption of the intestinal barrier, endotoxins, particularly lipopolysaccharide (LPS), translocate to the liver and activate intrahepatic immune cells *via* the portal venous circulation (46, 47). Hyperactivation of KCs through upregulated STING signaling leads to excessive pro-inflammatory cytokine production, while deficiency in PD-1 signaling enhances the bacterial clearance capacity of KCs (48, 49). In addition to modulating immune responses, bacterial toxins directly damage hepatocytes by activating programmed cell death pathways. Specifically, cytosolic LPS triggers caspase-11-mediated hepatocyte pyroptosis, a process inhibited by HSPA12A through PGC-1α-dependent regulation of AOAH expression (50).

Liver sinusoidal ECs (LSECs) are characterized by highly fenestrated structures and the absence of a basement membrane, making them a key interface for material exchange between hepatic sinusoids and hepatocytes. Additionally, LSECs serve as platforms for immune cell activation during inflammatory processes (51, 52). However, exposure to LPS and other pathogen-associated molecular patterns (PAMPs) during sepsis compromises the integrity of LSECs, resulting in sinusoidal capillarization and impaired endothelial autophagy (53, 54). The loss of fenestrations in LSECs disrupts sinusoidal perfusion and promotes excessive immune cell infiltration, thereby exacerbating oxidative stress and inflammatory cascades. In this study, Bud appears to preserve the structural and functional integrity of hepatic sinusoids by reducing the proportions of inflammatory Cox8a^+^Endo and Ccl2^+^Cxcl1^+^Endo subpopulations. Furthermore, the upregulation of Selenop in these subpopulations may exert antioxidant effects, alleviating oxidative stress in hepatic tissues (55). Previous studies have shown that LPS or interferon-γ stimulates LSECs to release chemokines that recruit pro-inflammatory immune cells and amplify local inflammation (56). P-selectin (Selp), an early mediator of acute inflammation expressed on activated ECs and platelets, interacts with P-selectin glycoprotein ligand-1 (PSGL-1) on neutrophils, monocytes, and effector lymphocytes to initiate leukocyte rolling and adhesion (34, 57). The Bud-mediated downregulation of Selp expression may inhibit neutrophil recruitment and subsequent inflammatory liver injury. Consequently, therapeutic strategies targeting LSECs could restore their immune regulatory function and help mitigate liver damage.

As key effectors of innate immunity, neutrophils play a central role in the pathogenesis of SALI through mechanisms such as degranulation to release antimicrobial granule components and the formation of NETs (58). NETs are net-like structures composed of granular proteins, such as neutrophil elastase, myeloperoxidase, and cytoplasmic proteins, attached to decondensed chromatin fibers (59). NET formation is significantly elevated during sepsis, and its excessive accumulation strongly correlates with enhanced inflammatory responses and organ dysfunction (60). This association is supported by two key studies. Cho et al. demonstrated that excessive NET production contributed to alcohol-associated liver damage, while Bukong et al. showed that abnormal NET formation and impaired NET clearance exacerbated sepsis-associated liver injury (61, 62). In addition to NET formation, neutrophil recruitment and migration are critical processes in liver injury. Macrophages promote neutrophil infiltration into the liver by activating the CXCL2-CXCR2 axis, which in turn stimulates NET formation through the secretion of pro-inflammatory cytokines such as IL-1β and TNF-α. This mechanism has been shown to exacerbate liver injury in models of age-related liver injury (63). In contrast, our findings suggest that Bud significantly inhibits CXCL2-CXCR2 signaling between ECs and neutrophils, potentially limiting neutrophil migration and alleviating NET-mediated liver injury. Notably, exogenous administration of Dnase1 and Dnase1l3 can degrade the DNA scaffold of NETs, mitigating their harmful effects in inflammatory diseases (64–66).

Although this study advances our understanding of Bud’s therapeutic potential against SALI, several limitations warrant further investigation. The 24-hour observation period following SALI model establishment may be insufficient to fully evaluate the long-term therapeutic effects of Bud, particularly in the context of chronic inflammation. Future studies should extend the observation period to assess both the sustained efficacy and potential adverse effects of Bud. While scRNA-seq provides valuable insights into Bud’s regulatory effects on the three key cell types, the precise molecular mechanisms involving signaling pathways and transcription factors remain incompletely understood. Functional validation through gene knockout, overexpression, or pharmacological inhibition is necessary to confirm the biological relevance of these findings. Furthermore, translating these results to clinical applications poses challenges due to the physiological and pathological differences between mice and humans. Additional preclinical studies in alternative animal models, as well as human clinical trials, are essential to evaluate the safety and efficacy of Bud in clinical settings.

## 5 Conclusion

In conclusion, this study provides insights into the mechanisms by which Bud effectively alleviates liver damage caused by SALI through the regulation of multiple cell types within the hepatic microenvironment and their intercellular interactions, providing a theoretical foundation for the development of natural product-based immunotherapeutic strategies.

## Data Availability

The datasets presented in this study can be found in online repositories. The names of the repository/repositories and accession number(s) can be found in the article/**Supplementary Material**.
